# Histopathology of Celiac Disease. Position Statements of the Italian Group of Gastrointestinal Pathologists (GIPAD-SIAPEC)

**DOI:** 10.37825/2239-9747.1005

**Published:** 2020-10-01

**Authors:** V Villanacci, C Ciacci, T Salviato, G Leoncini, L Reggiani Bonetti, T Ragazzini, F Limarzi, L Saragoni

**Affiliations:** 1 Institute of Pathology ASST-Spedali Civili, Brescia, Italy; 2 Celiac Center, AOU San Giovanni di Dio e Ruggi di Aragona, University of Salerno, Department of Medicine, Surgery, and Dentistry Scuola Medica Salernitana, Salerno, Italy; 3 Department of Diagnostic, Clinic and Public Health Medicine, University of Modena and Reggio Emilia, Modena, Italy.; 4 Pathology Unit, ASST del Garda, Desenzano del Garda, Brescia, Italy.; 5 Department of Pathology, University of Bologna, Italy.; 6 Department of Pathological Anatomy, AUSL Romagna, Morgagni-Pierantoni Hospital, Forlì, Italy

**Keywords:** histopathology, celiac disease, non-celiac gluten sensitivity, refractory celiac disease, ulcerative jejunitis, enteropathy-type T-cell

## Abstract

Celiac Disease (CeD) is an immune-mediated inflammatory disorder of the small intestine, affecting genetically susceptible individuals when exposed to gluten. Small intestinal biopsy interpretation has been the “gold standard” for celiac disease (CeD) for over 50 years. Despite today’s availability of sensitive and specific serological tests, the histopathological features from mucosal biopsy play a key role in diagnosing when CeD is suspected. Such a diagnostic approach requires a multidisciplinary team to optimize both tissue sampling and interpretation via the interaction between the pathologist and the gastroenterologist. Pathologists of the Italian Group of Gastrointestinal Pathology (GIPAD-SIAPEC), together with a member (TR) of the Italian Society of Technicians (AITIC) and an expert gastroenterologist (CC), provide position statements as a practical tool for reading and interpreting the report.

Moreover, a position statement was formulated about the recently described condition known as Non-Celiac Gluten Sensitivity (NCGS). Within such a diagnostic setting, both the architectural abnormalities of the duodenal mucosa, namely glandular hyperplasia, and villous atrophy and the number of intraepithelial T-lymphocytes should be well highlighted. Ancillary tests such as anti-CD3 stain are useful for an accurate count of the intraepithelial T lymphocytes when CeD or NCGS is suspected. Moreover, anti-CD3 and anti-CD8 stains are recommended in patients not responding to the gluten-free diet (GFD) to confirm a diagnosis of Refractory Celiac Disease (RCeD). Diagnostic clues about the differential diagnosis of both CeD and RCeD have also been rendered.

## I. INTRODUCTION

Celiac disease (CeD) is an immune-mediated inflammatory disorder of the small intestine, affecting genetically susceptible individuals when exposed to gluten [1]. Although sensitive and specific serological tests are nowadays available, a multidisciplinary approach to the clinical, serological, genetic, and histological features is recommended for the diagnosis of CeD. The prevalence of CeD is actually estimated to range from 0,2 to 1 worldwide, but it still remains largely under diagnosed [2–3] or diagnosed with a significant delay [4–7]. The growing shreds of evidence about diagnostic problems and pitfalls make necessary the formulation of position statements about the interpretation of the microscopic report, as to offer a practical and useful tool for pathologists and the non-specialized physicians. The major diagnostic hallmarks are here discussed and reviewed by a selected group of pathologists belonging to the Italian Group of Gastrointestinal Pathology (GIPAD-SIAPEC), with the collaboration of both an expert gastroenterologist (CC) and a member (TR) of the Italian Society of Technicians (AITIC), in order to define diagnostic key-points to provide a thorough histopathological report.

## II. METHODOLOGY

The authors have reviewed the available literature about CeD diagnosis, using the MeSH Terms “anatomy and histology,” “duodenum,” and “celiac disease” and/or “diagnosis.” The research produced 1323 papers, of which 984 according to the aim of the present study.

After the selection of the English language, and the exclusion of commentaries and meeting abstracts, the Authors evaluated 630 papers. Finally, they selected 60 papers, which included some recent guidelines that formed the bibliographic core of our study. The methodological approach to duodenal biopsy, the currently available serological and genetic tests, the histological features of both healthy and pathological duodenal mucosa, the differential diagnosis of CeD and its complications were critically reviewed in several meetings and teleconferences. As a result, the methodological approach to duodenal biopsy was summarized in eight position statements about the serological and genetic test records accompanying the samples, the histological features of both healthy and pathological duodenal mucosa, the differential diagnosis and complications of CeD. Moreover, the current knowledge about NCGS histology was also reviewed. The evidence levels of eight position statements were graduated according to the Guidelines of the Oxford Center for Evidence-Based Medicine (Oxford UK) and were discussed by all the working parties.

## III. RESULTS

Table 1 shows a synoptic view of the eight position statements.

### STATEMENT 1. A methodological approach to duodenal biopsy

**At least six mucosal biopsies are recommended, and biopsy orientation is strongly encouraged in order to avoid diagnostic pitfalls. [Grade of Evidence: 2]**

**Fig. 1:**
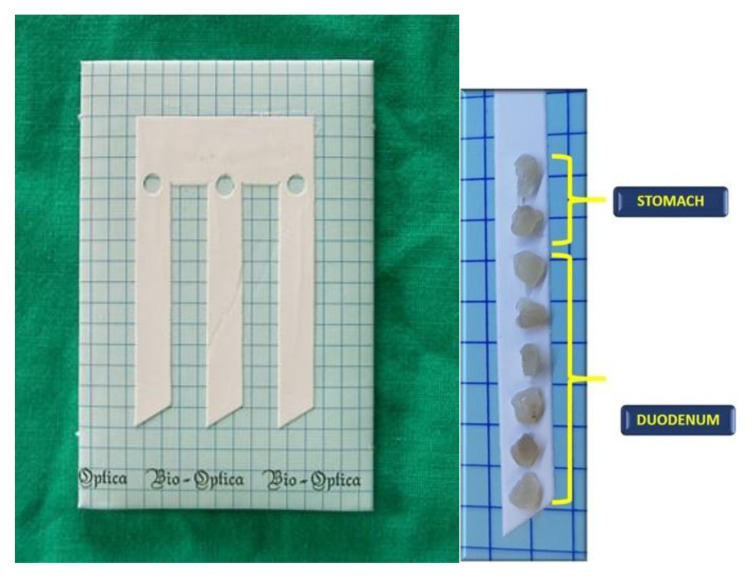
An example of cellulose acetate filters with a “clarinet beak-shaped cut. The adequate number of oriented biopsies of the duodenum and stomach on the filter.

Patients with familiarity, previous diagnosis of CeD [8], or clinical evidence of CeD [9,10] usually undergo an endoscopic evaluation with duodenal mucosa biopsies. However, it is not to be excluded that a routine endoscopy could recognize duodenal mucosal damage when CeD is clinically not suspected [11]. A correct evaluation of the mucosal damage should take I into account whether at the time of endoscopy, the diet regimen of the patient is free or not [12]. At least four to six mucosal pinch biopsies (2 from the bulb and 4 for the distal duodenum) are recommended to avoid diagnostic pitfalls or, at least, a reduced sensitivity, particularly in children (Figure 1 A and B).

**Fig. 2:**
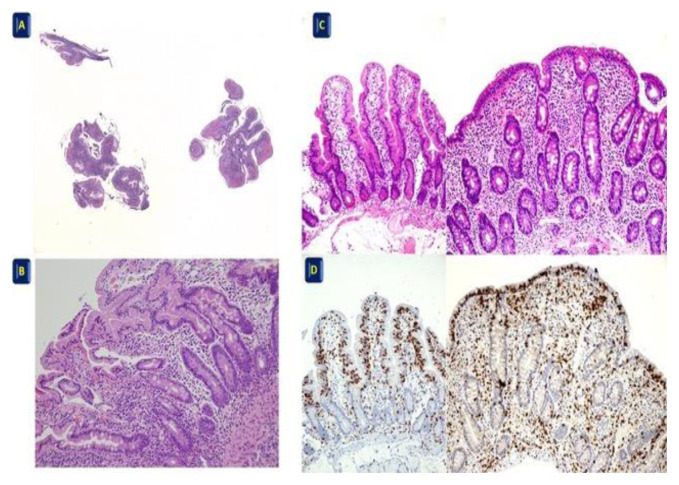
A-B Non oriented biopsies H&E A 4X, B 20X; C-D Oriented biopsies: here, it is possible to distinguish real atrophy and count the real number of IELs (C, H&E 10X and D, CD3 10X).

During the endoscopy, a single pinch biopsy for any passage is recommended [1–13,14,15,16]. Biopsy orientation could be relevant for a proper histological assessment, although no widely validated methods are accepted yet. Moreover, the application of this method requires endoscopists and endoscopic staff motivated and aware of the purposes of the method as well as an expert laboratory technician on the different steps necessary in order to reach optimal workout. In our experience, we found helpful using cellulose acetate filters with a “clarinet beak-shaped cut” (Fig. 1) because they guarantee the correct orientation of the biopsies during all phases of the sampling preparations (Fig. 2).

### STATEMENT 2. Serological and genetic tests

**The record of specific CeD serology, if known, should preferably accompany the histologic sample. The detection of serum anti-tissue transglutaminase IgA (TTGA) titer + IgG is the recommended serological test for screening/case finding. The anti-endomysial IgA search (EmA) is considered as a confirmatory test, and its determination is necessary for patients with low (<2 x) titer TTGA. The detection of anti-gliadin antibodies (AGA) titer together with negative TTGA and EmA titers never qualifies CeD in adult patients and in children. The detection of serum anti-deamidated gliadin peptides**

**(DPG) IgA and IgG may also be useful, especially in very young children. The detection of the IgG class of TTG EmA and DPG should be limited to patients with selective IgA deficiency. The genetic test for HLA DQ2-DQ8 supports the multidisciplinary diagnosis of CeD in selected cases, and if negative, it strongly excludes the diagnosis of CeD. [Grade of Evidence: 3]**

Availability of a serology report will boost the pathologists to the full description of intestinal mucosa findings.

In brief, IgA class anti-transglutaminase (TTGA) antibodies have the highest sensitivity for CeD (98%) with an estimated specificity of about 90%. IgA class anti-endomysium antibodies (EmA), although presenting a lower sensitivity compared to the IgA class TTGA (90% vs. 98%), show an absolute specificity for CeD. However, IgA anti-gliadin antibodies (AGA) are now an obsolete test with lower sensitivity and specificity for CeD.

The genetic tests play a role in supporting the diagnosis of CeD, for the association of the disease with the histocompatibility antigens HLA DQ2-DQ8. The genetic test is indicated when the serological and histological data are discrepant, in first degree relatives for the evaluation of genetic predisposition to CeD. The main clinical role of the genetic test in the diagnosis, however, is to exclude CeD when HLA-DQ2-DQ8 alleles are absent [8,9,10,11,12,13].

### STATEMENT 3. The healthy duodenal mucosa

**The healthy duodenal mucosa is characterized by a villus/crypt ratio of more than 3/1. An amount of less than 25 intra-epithelial lymphocytes (IELs)/100 epithelial cells have to be considered not pathological. [Grade of Evidence: 2]**

The healthy duodenal mucosa is characterized by folds, in which digitiform structures (villi) and pits (crypts) alternate, with a villus/crypt ratio of more than 3/1. In the lamina propria, a bland inflammatory infiltrate, composed by lymphocytes, plasma cells, eosinophils, histiocytes, mast cells can be found. Neutrophils are generally absent, with the exception of the active duodenitis with gastric metaplasia, related to Helicobacter Pylori (HP) infection. Lymphocytes may be seen forming scattered lymphoid aggregates in the lamina propria as well as within epithelial cells of the duodenal mucosa, i.e., intraepithelial lymphocytes (IEL). The presence of eosinophils, not exceeding 5/HPF, is not considered a pathological finding. The IELs count is a diagnostic key-point. The finding of more than 25 IELS/100 enterocytes should be considered unequivocally pathological, even in the regular duodenal mucosa, suggesting early CeD. In these cases, the use of CD3 immunostaining could be useful to avoid misdiagnoses, allowing the more accurate count of T intraepithelial lymphocytes. The CD8 immunostaining could be useful in the elderly patients, when a refractory celiac disease (RCeD) is suspected [14,15,16,17,18,19,20,21–23].

### STATEMENT 4. The pathological duodenal mucosa

**The histopathological features most commonly found in CeD are villous atrophy, crypts hyperplasia, increased number of IELs (25/100 epithelial cells). The IELs count must be performed both in the apical portions and along the side of the villi, incorrectly oriented biopsies with aligned epithelial cells and using an anti-CD3 monoclonal antibody. We strongly recommend the use of the classifications by Marsh and Corazza-Villanacci to improve the standardization of the terminology. [Grade of Evidence: 1]**

The histopathological features of the duodenal mucosa in the setting of CeD were classified by Marsh [24] with a subsequent modification by Oberhuber [25]. However, a modern consensus established that a cut-off of 25 IEL/100 enterocytes optimizes discrimination between normal control and CeD biopsies [26]. To standardize the terminology and to improve the diagnostic reproducibility, a new histological classification has been proposed by Corazza and Villanacci [27,28]. The two classifications are summarized and compared in Table 2. Recently a simplified classification with only two entities was proposed [29]

### STATEMENT 5. The histology report

**The Authors recommend listing the pathological features found in the duodenal mucosa in the histology report, avoiding the terms “celiac disease,” “gluten sensitivity/intolerance,” “malabsorption.” The use of anti-CD3 immunostain is strongly advised, in particular, in the non-atrophic cases. The use of ambiguous terminology is strongly discouraged. [Grade of Evidence: 3]**

CeD diagnosis results from an overall clinical, serological, and pathological assessment. The histology report should provide a comprehensive description of the duodenal mucosal lesions. It could be a descriptive report, summarizing the microscopic findings with a final diagnostic interpretation, or it could alternatively be in the check-list format [30]. Regardless of the report type, the pathological features should be listed, the terminology should be straightforward, the terms ‘celiac disease’ or lesion compatible with malabsorption/gluten sensitivity’ avoided, as they may be misleading. Atrophy should be graded, if present, as mild, moderate, and severe. The IELs count is a diagnostic key-point. A number greater than 25/100 epithelial cells is considered pathological. In the early phase of the disease, when the villi are present, the presence of a pathological amount of IELs, without architectural abnormalities in the duodenal mucosa, could be the only feature suggesting CeD. Thus, we recommend performing a CD3 immunostain. Application of the CD8 antibody could be useful in elderly patients when a refractory celiac disease (RCeD) is suspected [31,32].

### STATEMENT 6. The differential diagnosis

**Several clinical conditions share histopathological features with CeD, most of all, the increased IELs count. Thus, we strongly recommend a careful examination of the clinical setting. [Grade of Evidence: 2]**

A condition of hypersensitivity to non-gluten components of foods, including cereals, cow’s milk, soy products, fish, rice, and chicken, may be associated with increased IELs in affected patients, without villous atrophy. In some infections, such as in the Helicobacter Pylori-related gastritis [33,34], Giardia Lamblia, or Cryptosporidium, the duodenal mucosa shows an increased number of IELs without architectural abnormalities. Moreover, several drugs and autoimmune disorders produce the same histology findings [35]. Other reported conditions associated with an increased number of IELs include Hashimoto thyroiditis, Graves’ disease, rheumatoid arthritis, psoriasis, multiple sclerosis, and systemic lupus erythematosus. Common variable immune deficiency also causes intestinal mucosal damage due to inflammation and/or infections [36].

Furthermore, chronic inflammatory bowel diseases and collagenous and lymphocytic colitis have been concurrently associated with proximal small intestinal intraepithelial lymphocytosis. Noteworthy, graft versus host disease (GVHD) and other GVHD-like conditions show an increased IELs count. However, the clinical setting, the co-existence of both epithelial cell apoptosis, and some degree of architectural disturbance in GVHD allow proper microscopic interpretation [37]. In the enteropathy-type T-cell lymphoma (ETTL), neoplastic cells can be seen within a mildly atrophic or non-atrophic duodenal mucosa during the pre-infiltrative (*cryptic*) phase [38–41]. Flow-cytometry evaluation for <gamma>/<delta> IELs may help differentiate gluten-from non-gluten dependent conditions.[42]

### STATEMENT 7. The refractory celiac disease

**RCeD requires that a diagnosis of CeD has been already rendered, entailing a subsequent gluten-free diet. We recommend performing immunostains for CD3 and CD8 to differentiate RCeD1 from RCeD2 in biopsy samples taken when the patient is on a strict GFD. The use of the novel marker NKp46 could be considered. Further differential diagnosis includes other diseases mimicking CeD, such as autoimmune enteropathy and olmesartan-associated enteropathy. [Grade of Evidence: 3]**

Patients not responding to the gluten-free diet after 12 months may be suffering from RCeD. Two types of RCeD have been described. In equivocal cases, a second endoscopy and several biopsies are mandatory. The small bowel lesions in RCeD1, as well as in RCeD2, can be included in the Marsh classification criteria, with the prevalence of Marsh lesion type III, although Marsh lesion type II is possible. The presence of sub-epithelial collagen formation (similar to that seen in collagenous sprue), extending into the lamina propria with entrapment of capillaries or other cellular elements, the increased sub-cryptal chronic inflammatory cells, and mucosal atrophy with crypt hypoplasia are useful microscopic criteria for the diagnosis of RCeD [43,44]. The presence of aberrant IELs immunophenotype in RCeD2 differentiated in RCeD1 from RCeD2. Indeed, RCeD1 shows the same immunophenotype seen in CeD, with the majority of lymphocytes expressing CD3, CD7, CD8, CD103, and TCRβ. On the other hand, RCeD2 expresses CD103, CD7, and cytoplasmic CD3, but not surface CD3, CD4, CD8, or TCR-β. [45,47]. A diagnostic biomarker NKp46, belonging to the NK receptors (NKRs), has been recently proposed to differentiate RCD2 from RCD 1 since it was found to be significantly more expressed by malignant RCD2 IELs than normal IELs in CeD and RCD1 [48]. Some histopathological features consistent with RCeD are shared by other pathological conditions, such as the autoimmune enteropathy, a rare disease having some overlap with CeD, and olmesartan-associated enteropathy (an angiotensin II receptor blocker). The latter may be associated with a severe sprue-like enteropathy [49-50-51]. The clinical course of CeD can be complicated by further pathological conditions, namely ulcerative jejunitis (UJ) and ETTL, affecting the clinical outcome and the overall survival. UJ is a rare disease shown to evolve from pre-existing RCeD. Generally, the ulceration extends through the full thickness of the mucosa, with secondary vascular changes at the ulcer base. Coexistent chronic inflammation, fibrosis, and muscular hypertrophy, the latter responsible for the stricture formation, can be found. The non-ulcerated mucosa may display flattening, and villous atrophy along with other CeD-like changes, such as crypt hyperplasia, IELs infiltration, superficial enterocytes irregularity, and mixed infiltrate composed by plasma cells, eosinophils, and neutrophils, both adjacent to-and remote from-areas of ulceration. Transmural inflammation and submucosal edema are occasional, but lymphoid follicles, granulomas, or giant cells are usually absent. RCeD histological and immunohistochemical features may also be seen [52–53]. UJ may evolve within the background of RCD as full-thickness ulceration of mucosa surrounded by villous atrophy and CD-like changes. ETTL is assumed to derive from IELs, and the aberrant immune phenotype seen in RCeD2 IELs represents an early stage in the development of overt lymphoma. Two distinct histological subtypes have been recognized. Type 1 ETTL (ETTL-1) shows an infiltrate of medium-sized cells containing round or angular nuclei with prominent nucleoli and a moderate amount of eosinophilic cytoplasm. In some cases, the tumor cells may display marked pleomorphism, recalling anaplastic large-cell lymphoma or Hodgkin’s lymphoma. Type 2 ETTL (ETTL-2) is rare and comprises a monomorphic population of small cells with hyperchromatic nuclei and minimal cytoplasm. In the intact/non-tumor mucosa, features of CeD can be seen, including intraepithelial lymphocytosis. The tumor cells in ETTL-1 express CD3 and CD7, but not CD4, CD8, CD5, or CD56. The cells with an anaplastic morphology show CD30 positivity. The IELs in the non-neoplastic mucosa have the same immunophenotype as in RCeD2, UJ, and ETTL-1 (CD3+, CD4− / 8−, CD56−). In contrast, the neoplastic cells in ETTL-2 show a CD3+, CD8+, CD56+, CD4− pattern, and this profile is also seen in the majority of adjacent IELs, with only a minor CD4−/CD8− population [51,52,53,54,55,56,57]. NKp46 was also detected in ETTL, highlighting its progression from RCD2 [48].

### 3.8. STATEMENT 8. Non-Celiac Gluten Sensitivity

**The Non-Celiac Gluten Sensitivity (NCGS) has been associated with duodenal biopsies showing normal villi, increased eosinophils in the lamina propria, and normal IELs count, but with both a peculiar lymphocytic arrangement in small intra-epithelial clusters and a linear disposition in the deeper mucosa. In such instances, a thorough clinical-pathological correlation is strongly recommended. [Grade of Evidence: 3]**

The histologic characteristics of NCGS are still under investigation, ranging from normal histology to a slight increase in the number of T lymphocytes in the superficial epithelium of villi. Some authors describe a normal number of T lymphocytes but a peculiar disposition of this cells in a small “cluster” of 3–4 elements in the superficial epithelium, as well as the linear disposition in the deeper part of the mucosa together with an increased number of eosinophils (>5/HPF) in lamina propria. Further studies are needed to assess these findings as specific for NCGS [58,59,60].

## Figures and Tables

**Table 1:** Main topics and statements for a correct gluten intolerance diagnosis

	TOPICS	STATEMENTS
1	Methodological approach to biopsy	At least four mucosal biopsies are recommended, and biopsy orientation is strongly encouraged in order to avoid diagnostic pitfalls.
2	Serological and genetic tests	The detection of TTGA titer (plus AGA in children younger than 2 years) is recommended. The detection of AGA titer together with negative TTGA and EmA titers never qualify CeD in adult patients and in children older than 2 years. The detection of the IgG class should be limited to patients with selective IgA deficiency. The genetic test could support the multidisciplinary diagnosis of CeD in selected cases.
3	Healthy duodenal mucosa	The healthy duodenal mucosa is characterized by a villus/crypt ratio more than 3/1. A lymphocytic amount of more than 30 lymphocytes/100 epithelial cells has to be considered as pathological. The IELs count must be performed both in the apical portions and along the side of the villi, in perfectly oriented biopsies with aligned epithelial cells and using anti-CD3 monoclonal antibody.
4	Pathological duodenal mucosa	We strongly recommend the use of the classifications by Marsh and Corazza-Villanacci in order to improve the standardization of the terminology.
5	The histology report	We suggest to list the in the histology report all pathological features observed in the duodenal mucosa consisting with Ced.
6	Differential diagnosis	Several clinical conditions share some histopathological features with CeD, most of all the increased IELs count. Thus, we strongly recommend a careful examination of the clinical setting.
7	Refractory Celiac Disease	RCeD requires that a diagnosis of CeD has been already rendered, entailing a subsequent gluten-free diet. We recommend performing immunostains for CD3 and CD8 in order to differentiate RCeD1 from RCeD2. The use of the novel marker NKp46 could be considered. A further differential includes other disease mimicking CeD, such as autoimmune enteropathy and Olmesartan-associated enteropathy.
8	NCGS	The NCGS may be suspected in duodenal biopsies characterized by normal villi, increased eosinophils in the lamina propria and normal IELs count, but with both a peculiar lymphocytic arrangement in small intra-epithelial clusters and a linear disposition in the deeper mucosa. In such instances, a thorough clinical-pathological correlation is strongly recommended.

**Table 2:** comparison among the current main classifications of mucosal damage in CeD.

Marsh mod. Oberhuber		Corazza-Villanacci	Villanacci
Lesions	Diagnostic Criteria	Lesions	Lesions
**Type I lesion** ***infiltrative***	No architectural changes **(villous/cript ratio preserved)** Increased IELs count **(> 25/100 epithelial cells)**	**Grade A lesion not atrophic** **No architectural changes (villous/cript ratio preserved)** **Increased IELs count (> 25/100 epithelial cells)**	**A Non atrophic type** No architectural changes **(villous/cript ratio preserved)** Increased IELs count **(> 25/100 epithelial cells)**
**Type II lesion** ***hyperplastic***	No architectural changes **(villous/cript ratio preserved)** Crypt hyperplasia **(mitoses > 1/crypt)** Increased IELs count **(> 25/100 epithelial cells)**		
**Type III A lesion** ***destructive***	Villous atrophy (mild degree) Crypt hyperplasia **(mitoses > 1/crypt)** Increased IELs count **(> 25/100 epithelial cells)**	**Grade B1 lesion*partial atrophy*** Villous atrophy (milsmoderate degree) Crypt hyperplasia **(mitoses > 1/crypt)** Increased IELs count **(> 25/100 epithelial cells)**	**B Atrophic Type** Villous atrophy (mildmoderate-severe degree) Crypt hyperplasia **(mitoses > 1/crypt)** Increased IELs count **(> 25/100 epithelial cells)**
**Type III B lesion** ***destructive***	Villous atrophy (moderate degree) Crypt hyperplasia **(mitoses > 1/crypt)** Increased IELs count **(> 25/100 epithelial cells)**		
**Type III C lesion** ***destructive***	Villous atrophy (severe degree) Crypt hyperplasia **(mitoses > 1/crypt)** Increased IELs count **(> 25/100 epithelial cells)**	**Grade B2 lesion*total atrophy*** Villous atrophy (severe degree) Crypt hyperplasia **(mitoses > 1/crypt)** Increased IELs count **(> 25/100 epithelial cells**	

## ABBREVIATIONS

CeD: celiac disease
NCGS: non-celiac gluten sensitivity
RCeD: refractory celiac disease
UJ: ulcerative jejunitis
ETTL: enteropathy-type T-cell lymphoma
TTGA: anti-transglutaminase antibodies
EmA: anti-endomysium antibodies
AGA: anti-gliadin antibodies
IELs: intra-epithelial lymphocytes
HP: Helicobacter Pylori
GFD: gluten free diet
NKRs: NK receptors
HPF: high power fields.
